# In-Plane Impact Response of Graded Foam Concrete-Filled Auxetic Honeycombs

**DOI:** 10.3390/ma16020745

**Published:** 2023-01-12

**Authors:** Xiaojuan Wang, Kuncheng Jia, Yan Liu, Hongyuan Zhou

**Affiliations:** 1 Key Laboratory of Urban Security and Disaster Engineering of Ministry of Education, Beijing University of Technology, Beijing 100124, China; 2 College of Civil and Architectural Engineering, North China University of Science and Technology, Tangshan 063210, China; 3 State Key Laboratory of Explosion Science and Technology, Beijing Institute of Technology, Beijing 100081, China

**Keywords:** auxetic honeycomb, foam concrete, impact mitigation, energy absorption, structure protection

## Abstract

Foam-filled honeycombs have been widely applied due to their excellent load transfer mitigation and energy absorption capacity. In the present study, a layered graded foam concrete-filled auxetic honeycomb was proposed by tuning its overall compression deformation mode to layer-by-layer deformation mode to realize multi-level structural protection. The effect of the honeycomb cell-wall thickness gradient (with an average thickness of 0.25 mm, thickness gradients of 0.30:0.25:0.20, 0.35:0.25:0.15 and 0.40:0.25:0.10, and corresponding positive gradients) and the foam concrete filler density gradient (408:575:848, 848:575:408) on the response mode, load transfer, energy absorption, and Poisson’s ratio of the proposed composite was systematically investigated. The results showed that the graded composite exhibited an obvious layered deformation mode and a negative Poisson’s ratio effect under relatively low and moderate loading rates (1 m/s, 10 m/s, respectively), especially with the foam concrete density gradient. Under a high loading rate (100 m/s), the graded composite demonstrated progressive collapse initiating from the loading end with a layer-by-layer crushing mode, regardless of the thickness and density gradient. In the response of the composite with a 0.2:0.2:0.2 thickness ratio and a 408:575:848 foam concrete gradient subjected to 1 m/s crushing, the first-layer, second-layer, and third-layer foam concrete absorbed 94.62%, 88.72%, and 86.94% of the total foam concrete energy absorption in the corresponding crushing stage, respectively. Compared with the counterpart homogeneous composites, although the graded composite had an insignificant improvement on energy absorption (less than 5%), it was able to significantly reduce the peak load (as high as 30%) to mitigate the load transfer to the protected structure. The effective Poisson’s ratio of the first layer in the composite with positive gradient (408:575:848) increased to −2 then converged to −0.6 under 2 m/s and 10 m/s crushing, and ranged from −0.4 to −0.1 under 50 m/s and 100 m/s crushing, respectively. The effective Poisson’s ratio of the middle and bottom layers increased to −2 initially and converged to range −0.4 to −0.1, regardless of the crushing speed. The staged response mode of the graded composite facilitated the realization of multi-level structure protection with significantly reduced peak load transferred to the protected structure and tuned energy absorption.

## 1. Introduction

The accidental industrial explosions and terrorist attacks have posed significant threats to important structures and infrastructures. Therefore, understanding the response of structures is necessary to facilitate structure protection against extreme loading. The response of existing structures, especially reinforced concrete (RC) structures, subjected to blast [1,2], impact [3], and penetration [4] have been extensively and intensively investigated. Furthermore, iso-damage curves based on the pressure–impulse diagram have been established to describe the response and damage extent of the structures [5].

While damage of RC structures is almost inevitable when subjected to blast or impact, applying sacrificial cladding to the structure exterior is a promising protection method. Sandwich structures are widely applied in the aerospace, vehicle, ship, and packaging industries owing to their advantages of being lightweight, having a high specific strength, a strong crashworthiness, and a high energy absorption efficiency. In recent years, due to their excellent energy absorption capacity, they have received increasing attention in the field of structure protection [6,7,8,9,10]. In general, a sandwich structure is composed of solid face and back plates, and a lightweight cellular core. While the cellular core absorbs a considerable amount of energy, various types of cores with excellent energy absorption capacity have been proposed, for instance, metal foam core [11,12], polymeric foam core [13,14], foam concrete core [15,16,17], honeycomb core [18], negative Poisson’s ratio honeycomb core [19], foam-filled honeycomb core [20,21], and other high-performance cores [22]. The mechanical performance and energy absorption of these core materials or structures subjected to quasi-static and dynamic loads have been extensively and intensively investigated based on the approaches of experimental tests, numerical simulations, and theoretical analyses.

Impact and blast are two major strong dynamic loads, possibly leading to severe damage or strong vibration [23,24,25]. For the dynamic response of sandwich structures subjected to blast load, Fleck et al. [26] divided the entire response process into three stages, namely, fluid–structure interaction, core compression, and overall structural response. They observed that the energy absorption mainly occurred during the core compression stage. Zhang et al. [27,28] systematically investigated the dynamic response of sandwich beams and slabs subjected to impact load based on approaches of theoretical analysis and numerical simulation. The results showed that the sandwich beams or slabs could undergo large deformation, provide steady plateau stress, and absorb a considerable amount of impact energy. The sandwich core plays an important role in energy absorption efficiency since the core can absorb a large amount of energy through large deformation to resist impact or blast loads.

The foam materials and honeycomb are usually applied as the sandwich core due to their excellent energy absorption performance, but their individual use has exhibited unfavorable performances due to their relatively low plateau stress. To take advantage of foam materials and honeycomb, lightweight foam-filled honeycomb was proposed by a simple combination of these two components with significantly improved energy absorption capacity [29]. Therefore, the traditional hexagonal honeycomb has been widely applied in various foam-filled structures due to its advantages of a simple manufacturing process, strong impact resistance, and excellent energy absorption capacity [30,31,32]. The hexagonal honeycomb filled with foam concrete [33], expanded polypropylene foam [34], and polyurethane foam [35] were proposed, and thorough research on their mechanical performance and energy absorption was conducted. The results showed that the honeycomb could effectively constrain the lateral expansion of the filled foam while the filler provided effective support for the honeycomb; thus, the load-bearing capacity and energy absorption of the foam-filled honeycomb were significantly improved due to the interaction between honeycomb and filler compared with their individual use. For instance, the experimental and numerical study on the dynamic compression response of foam-filled multi-layer folded paper structure showed that the bearing capacity of foam-filled structure was higher than the summation of the bearing capacity of the individual foam material and multi-layer folded paper structure [36].

On one hand, due to the characteristics of outward expansion under uniaxial tension and inward shrinkage under uniaxial compression, the concave hexagonal negative Poisson’s ratio honeycomb has a higher yield strength [37], stronger energy absorption capacity [38], larger shear modulus [39], better stress diffusion effect, higher fracture toughness [40], and tunable mechanical properties [41] compared with the traditional hexagonal honeycomb. Thus some negative Poisson’s ratio structures have been proposed and examined. For instance, Zhang et al. [42] carried out theoretical analysis and numerical simulation on the deformation characteristics of hexagonal negative Poisson’s ratio honeycomb under tension. These results showed that the negative Poisson’s ratio structures exhibited excellent mechanical performance and energy absorption capacity, which has great potential to be applied as the sandwich core.

On the other hand, foam material, including polymeric foam, metal foam, and foam concrete, is an ideal filler with large densification strain and excellent energy absorption capacity. The polymer foam has the advantage of lightweight but the energy absorption capacity is comparatively unfavorable. Metal foam exhibits excellent energy absorption capacity, but it suffers from high cost and inconvenient construction. In comparison, foam concrete shows more potential to be applied in the field of structural protection due to its merits of being lightweight, low-cost, and easy to manufacture [43,44]. A series of uniaxial tension, uniaxial compression, and tri-axial compression tests on foam concrete were carried out [45,46,47,48,49], and the results showed that the bearing capacity and energy absorption of foam concrete were significantly improved by the lateral constraint. The performance of the foam concrete-filled honeycomb was experimentally and numerically investigated, and it was found that the foam concrete could greatly strengthen the collapse resistance of cell-wall [50]. Therefore, with the approach of filling foam concrete in a negative Poisson’s ratio honeycomb, the honeycomb can impose constrain on the foam concrete filler to improve its bearing capacity and energy absorption. Meanwhile, the foam concrete filler is beneficial to prevent premature bending, buckling, and shear failure of the honeycomb cell wall, as well as improve its bearing capacity owing to the wall-filler interaction.

It has been noted that the mechanical properties and energy absorption capacity of the foam material are mainly determined by its density, and the failure always initiates at the weakest parts which are somewhat random if the loading rate is lower than a certain value [51]. For the honeycomb with a regular structural architecture, its cell-wall thickness, relative density, and cell geometries are readily tailored for specified functions [52,53]. Therefore, the mechanical properties and energy absorption capacity of the foam-filled honeycomb heavily depend on the geometry and relative density of the cells. Although the homogeneous foam-filled honeycomb demonstrates stable mechanical properties, it is difficult to fulfill the diverse requirements of energy absorption [54,55,56]. To address this issue, with the determined cell form, an appropriate design of the relative density of the cell is the most straightforward way to achieve the aim of multi-level energy absorption. Cui et al. [57] studied the effect of density difference, average density, and impact energy of the gradient cellular structures on their energy absorption capacity. Liang et al. [58] adopted a Voronoi model to simulate continuous gradient cellular structures, and they found that a continuous gradient scheme was difficult to simultaneously improve the energy absorption and reduce the transmitted load to the protected structure under blast load. Furthermore, compared with the continuous gradient cellular structure, the layered gradient cellular structure is more promising owing to its merits of low cost, easy manufacture, and reliable mechanical properties. Avila [59] and Apetre et al. [60] studied the failure modes of sandwich structures with hierarchical gradient foam cores under quasi-static and dynamic loads, respectively, finding that the layered gradient sandwich structure could effectively reduce the damage caused by impact load. 

As to impact or collision mitigation, composites with soft core seem promising. Take the bridge pier collision mitigation in the field of civil engineering as an example. The composites can be used as protection cladding attached to the pier. If a vehicle out of control collides with the pier, the composite cladding could dissipate a large amount of energy of the system consisting of the pier, the vehicle, and the cladding, with major deformation concentrated in the cladding. At the same time, the loads to the pier as well as to the vehicle are significantly reduced. Consequently, both the pier and the vehicle with its occupants are effectively protected. After the incident, replacing the damaged cladding could immediately resume the protection capacity.

From the viewpoint of practical application, the protection performance could be improved if the gradient was introduced to the core, as the load (per unit area) transferred to the protected structure is the plateau stress of the crushed core. If the collision is moderate, the cladding response likely only occurs in the part of the core with the transferred load as the plateau stress of the crushed part, while the other part remains elastically deformed. Alternatively, in a severe collision, the gradient with direction could be designed to reduce the load transferred to the protected structure compared to that of the homogenous counterpart.

Among different foams being filled into hollow structures in the literature, foam concrete is promising in its application in civil engineering. On one hand, foam concrete is cost-effective and highly durable in typical service conditions such as water and oxygen. On the other hand, it can be conveniently cast into hollow structures of almost all typical geometries, which is more convenient than other foams needing cutting before filling, such as metallic foams. 

In the present study, layered gradient auxetic honeycombs filled with foam concrete are proposed, in which the gradient effect was realized by tuning the cell-wall thickness of the honeycomb and foam concrete density. The influence of the honeycomb cell-wall thickness gradient, the foam concrete density gradient, the loading rate on the response mode, load transfer, and energy absorption of the proposed composites was investigated with numerical models verified by test data. In addition, the effective Poisson’s ratio was employed to evaluate the concave deformation of each layer in the proposed gradient composites subjected to different loading rates.

## 2. Experimental Study

### 2.1. Specimen Preparation

**Figure 1:**
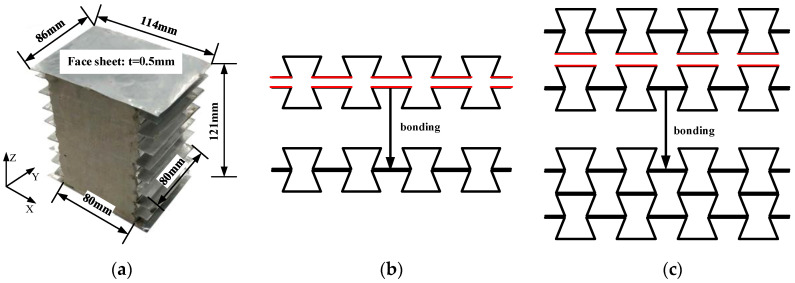
The auxetic honeycomb filled with foam concrete: (**a**) the specimen photo; (**b**) illustration of joining corrugated sheets to form auxetic honeycomb cells; (**c**) illustration of joining lines of cells to form auxetic honeycomb part. In (**b**,**c**), the edges in red were joined by adhesive.

The prepared foam concrete-filled auxetic reentrant honeycomb specimens are shown in Figure 1a, where the length, height, and out-plane width of the specimens were 80 mm, 120 mm, and 80 mm, respectively. As shown in Figure 1a, the specimen consisted of two different materials, namely, the aluminum honeycomb and the filled foam concrete. The aluminum sheet with a thickness of 0.25 mm was applied to fold the honeycomb, which can be regarded as a porous material with a relative density of 5.1%. From the illustration of the honeycomb preparation process in Figure 1b, the thickness of the oblique cell wall of the auxetic honeycomb core was 0.25 mm, while the thickness of the horizontal cell wall was 0.5 mm since the adjacent honeycomb cells were bonded together in layers with high-strength adhesive. In addition, two aluminum plates with a thickness of 0.5 mm were employed as the two face sheets, indicated in Figure 1a. There were seven staged honeycomb cells along the horizontal (x) direction and eight honeycomb cells along the vertical (z) direction. In addition to the hollow aluminum honeycomb, specimens filled with 408 kg/m^3^ foam concrete were also prepared for the quasi-static and dynamic compression tests.

It is worth noting that the dimension of the honeycomb was designed based on the loading capacity and the dimension of the crosshead and anvil of the INSTRON VHS160/100-20 high-speed testing system. On one hand, the crosshead and anvil were both round in shape with a diameter of 120 mm. Therefore, the honeycomb core was designed to be 80 mm* 80 mm to be within the area of the crosshead and anvil. On the other hand, with this specimen dimension, combined with the selected honeycomb material and thickness and typical foam concrete property, the crushing resistance was lower than the loading capacity of the high-speed testing system (100 kN), which was described in the following section.

### 2.2. Quasi-Static and Dynamic Compression Tests

**Figure 2:**
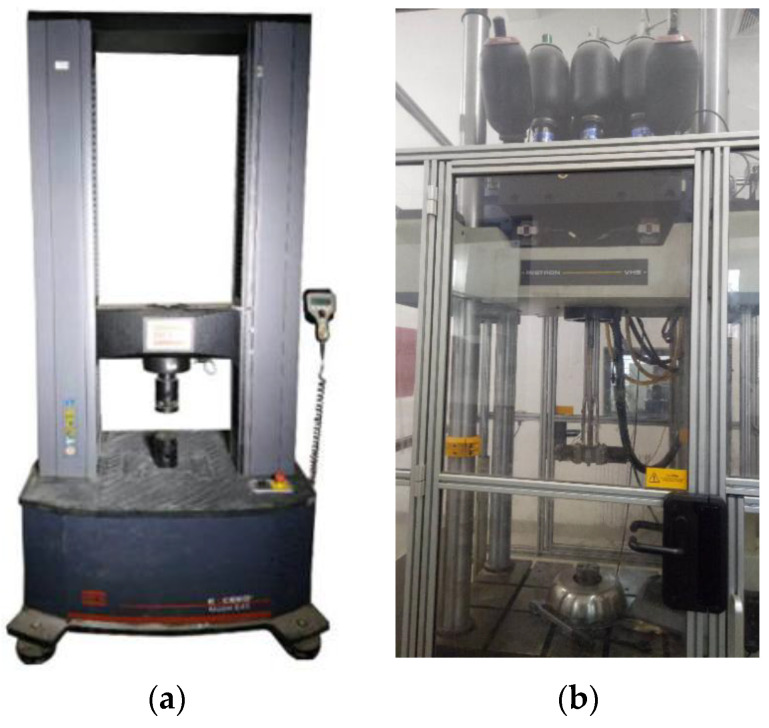
The testing machines used in the present study: (**a**) MTS Exceed E45 testing system; (**b**) INSTRON VHS160/100-20 high-speed testing system.

The quasi-static compression tests with a displacement control of 5 mm/min were conducted with an MTS Exceed E45 testing system with a loading capacity of 300 kN, shown in Figure 2a, and the compressive loading stopped when the composite reached its densification strain. Meanwhile, the dynamic tests with the compressive velocity of 1 m/s were carried out with an INSTRON VHS160/100-20 high-speed testing system with a loading capacity of 100 kN, shown in Figure 2b. It is known that foam concrete, as a typical cellular material, usually exhibits a sharp increase in bearing capacity from the stress plateau stage to the densification stage. Since the majority of materials in this composite are foam concrete, its bearing capacity is likely to rise rapidly after reaching its densification strain. Due to the limited loading capacity of the INSTRON high-speed instrument in the dynamic test as well as the rapidly raised bearing capacity of the composite after the stress plateau, the stress–strain curve of the densification stage of the composite in the dynamic test was not fully measured. The INSTRON VHS160/100-20 high-speed testing system was at Tianjin University, China. It was capable of compressing the specimen with a speed as high as 10 m/s. Its major advantage is that the machine can load (compression or tension) the specimen with almost constant speed regardless of the specimen resistance during loading, due to a rapid feedback mechanism. Consequently, a constant strain rate during loading can be roughly maintained. While the specimen height was not large, the compression speed could not be designed to be too high, otherwise, the buffer distance would be insufficient. To this end, 1 m/s compression was adopted to the hollow and filled auxetic honeycombs, to balance the loading speed on the specimen and the safety of the testing machine.

**Figure 3:**
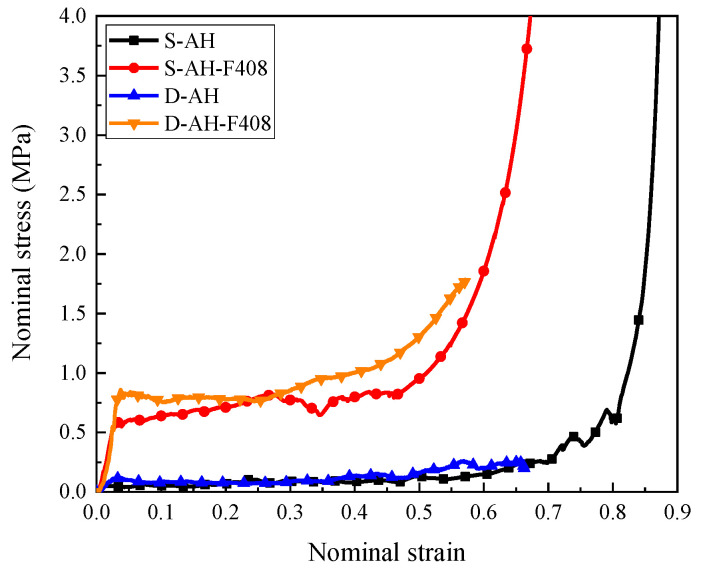
The stress–strain relationships of the specimens with different loading rates. The thickness of the honeycombs was 0.25 mm, for both the hollow and filled specimens. D in the sample index indicates the quasi-static and dynamic tests, respectively. AH represents auxetic honeycomb while F means foam concrete. For instance, D-AH-F408 represents the auxetic honeycomb filled with 408 kg/m^3^ foam concrete subjected to dynamic compression with 1 m/s.

Figure 3 demonstrates the measured stress–strain curves of hollow honeycomb and honeycomb filled with 408 kg/m^3^ foam concrete subjected to quasi-static and dynamic uniaxial compression, respectively. For the hollow aluminum honeycomb, there was a negligible difference in the measured stress–strain curves between the quasi-static compression and dynamic test except that the initial peak stress was slightly higher in the dynamic test. As shown in Figure 3, the hollow aluminum honeycomb in quasi-static and dynamic compression tests provided ra elatively low compression strength and plateau stress, since the cell wall exhibited a rapid in-plane compression deformation without filler. As shown in the stress–strain curves of the honeycomb filled with 408 kg/m^3^ foam concrete under quasi-static and dynamic compression in Figure 3, the foam concrete-filled honeycomb exhibited a significantly higher compressive strength and plateau stress due to the continuous support provided by the foam concrete filler, compared with the hollow honeycomb.

**Figure 4:**
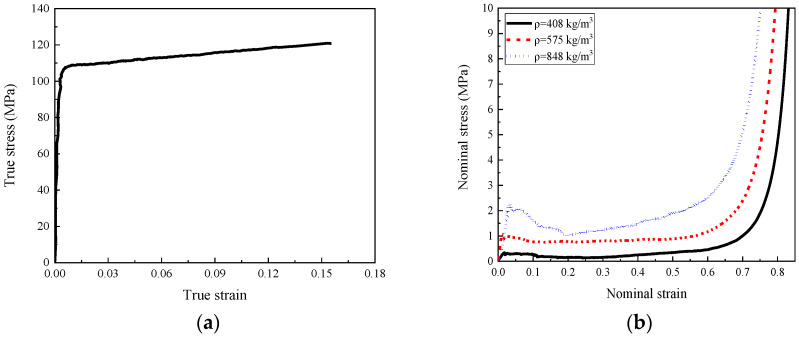
The stress–strain relationships under quasi-static loading: (**a**) coupon of honeycomb cell-wall material under tension of loading rate 0.5 mm/min; (**b**) foam concrete under compression of loading rate 0.5 mm/min.

In addition, with the loading rate of 0.05 mm/min, the quasi-static tensile test of the dog-bone aluminum sheet was carried out according to the standard ASTM E8M-04 to investigate the tensile mechanical properties of the cell-wall material, and the true stress–strain curve is shown in Figure 4a, which would be applied to define the stress–strain relationship of the honeycomb cell-wall in the numerical simulation. Furthermore, the quasi-static compression tests with a displacement control of 0.5 mm/min were carried out on the cubic foam concrete specimens with a size of 100 mm × 100 mm × 100 mm and different densities to investigate its mechanical properties, while the stress–strain relationships of foam concrete is presented in Figure 4b.

## 3. Numerical Model with Validation

### 3.1. Numerical Model of the Homogenous Composite

**Figure 5:**
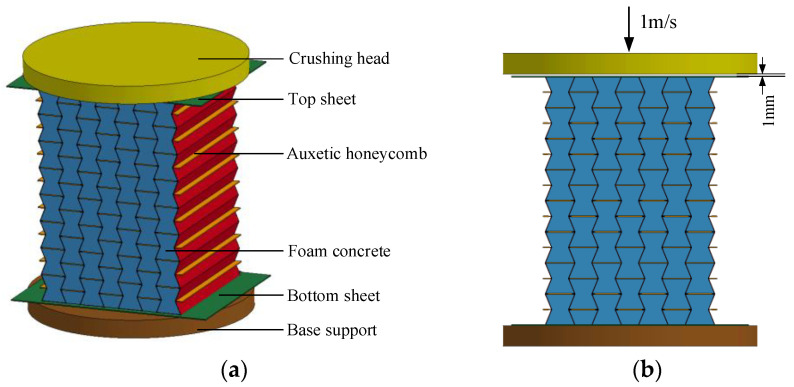
The numerical model: (**a**) model configuration; (**b**) loading mode.

The numerical simulation was carried out with the finite element software LS-DYNA 971. As solid structures with contact interaction were concerned in the present study, the Lagrangian approach was adopted to ensure computational accuracy. Specifically, in the specimen, the auxetic honeycomb, top sheet, and bottom sheet were typical thin-walled structures; therefore, they were modeled with Shell elements to balance computational cost and accuracy. Moreover, the foam concrete filled in the auxetic honeycomb was typically solid; thus, it was modeled with Solid elements. The numerical model fully represented the specimen geometry except for the shorter horizontal edges, which were designed slightly longer for adequate bonding in the test. In the numerical model, the foam concrete-filled auxetic honeycomb was sandwiched by the anvil and the crosshead. All degrees of freedom of the anvil, as well as all the rotations and the in-plane translations of the crosshead, were constrained. As shown in Figure 5, the crushing head was initially located 1 mm above the top flat face sheet of the specimen, and it moved along the out-of-plane direction with 1 m/s constant crushing velocity till the specimen reached the strain around 0.75.

As shown in the numerical model in Figure 5, the upper and lower face plates and the aluminum honeycomb core layer were modeled with Belytschko-Tsay shell element SHELL163. This shell element is a four-node element with bending and membrane characteristics; thus, it can withstand both in-plane and out-plane loads. There are six degrees of freedom at each node, namely, three translations and three rotations in the x-, y-, and, z-direction. As shown in Figure 1, the upper and lower aluminum face plates, as well as the middle aluminum concave folded plates were bonded together with LEAFTOP1160 high-strength epoxy resin during the manufacture of the aluminum honeycomb. In both the quasi-static and dynamic compression tests, no separation was observed between the adjacent aluminum plates during the entire loading process. Therefore, in the numerical model, it was assumed that the adjacent aluminum sheets were completely bonded together during the entire deformation process, and the thickness of the adhesive layer was neglected. Therefore, in this model, the thickness of the horizontal cell wall was set to 0.5 mm while the thickness of the oblique cell wall was defined as 0.25 mm. With tensile test data of the aluminum sheet in Figure 4a, the stress–strain curve was defined by the keyword *MAT_PIECEWISE_LINEAR_PLASTICITY to describe the material properties of the aluminum honeycomb cell wall and the two face plates. Moreover, since the strain rate sensitivity of 1060 aluminum alloy was insignificant, the strain rate effect was neglected in the material model. The material parameters of aluminum in the numerical simulation are listed in Table 1.

The crosshead and the anvil were simplified as a cylindrical base and loading plate, which were modeled by the eight-node solid element SOLID164. Since the strength and stiffness of the crosshead and anvil were significantly higher than that of the composite, the deformation of the crosshead and anvil was neglected in the numerical model. Therefore, the loading device, consisting of the crosshead and the anvil, was assumed to be rigid in this numerical simulation by the material model keyword *MAT_RIGID. The material properties of the filled foam concrete were defined by the keyword *MAT_CRUSHABLE_FOAM in the numerical model. This material model was a macroscopic equivalent model, and it was assumed that the foam concrete was an isotropic homogeneous material with the provided macroscopic mechanical properties. In this model, the mechanical properties of foam concrete were defined by the damping, tensile cut-off stress, and the measured stress–strain curve in the compression test in Figure 4b. The tensile cut-off stress (TSC) of foam concrete was set to 1/10 of the initial peak compressive stress [61,62]. It was worth noting that a small Poisson’s ratio value of 0.01 was applied for the foam concrete filler, which would inevitably underestimate its lateral expansion in the numerical results since the Poisson’s ratio would increase with increasing compression deformation. In addition, the strain rate effect of the foam concrete filler was considered by the parameter DAMP (damping coefficient), which was set to 0.1 in the present study. The material parameters of foam concrete in the numerical simulation were listed in Table 2.

The components in the auxetic honeycomb, including the inner concave folded plates, and the upper and lower surface plates, were connected through a common node contact. The automatic single-to-surface contact algorithm with the keyword *AUTOMATIC_SINGLE_SURFACE was applied to simulate the self-contact of auxetic honeycomb and foam concrete. Meanwhile, an automatic face-to-face contact algorithm with the keyword of *AUTOMATIC_SURFACE_TO_SURFACE was applied between the loading platens and the face sheets, auxetic honeycomb cell wall, and foam concrete filler. The static and dynamic friction coefficients were set to 0.3 and 0.2, respectively. Furthermore, the mesh size sensitivity study of the numerical model was carried out, and it was found that a 2 mm mesh size was a reasonable choice balancing the simulation accuracy and computational efficiency. In the model, around 680,000 elements are used.

### 3.2. Numerical Model Validation

Before investigating the response of the foam concrete-filled auxetic honeycomb subjected to impact, the numerical model was validated in terms of the nominal stress–strain curve for both hollow honeycomb and that filled with foam concrete, respectively.

#### 3.2.1. Hollow Honeycomb

**Figure 6:**
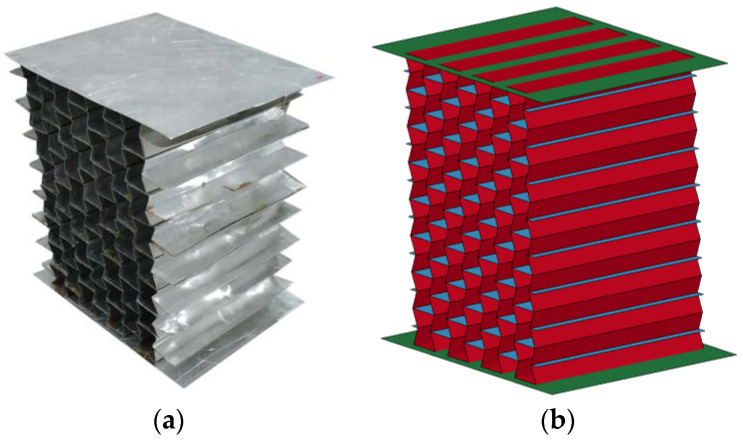
The hollow auxetic honeycomb: (**a**) photo; (**b**) numerical model.

**Figure 7:**
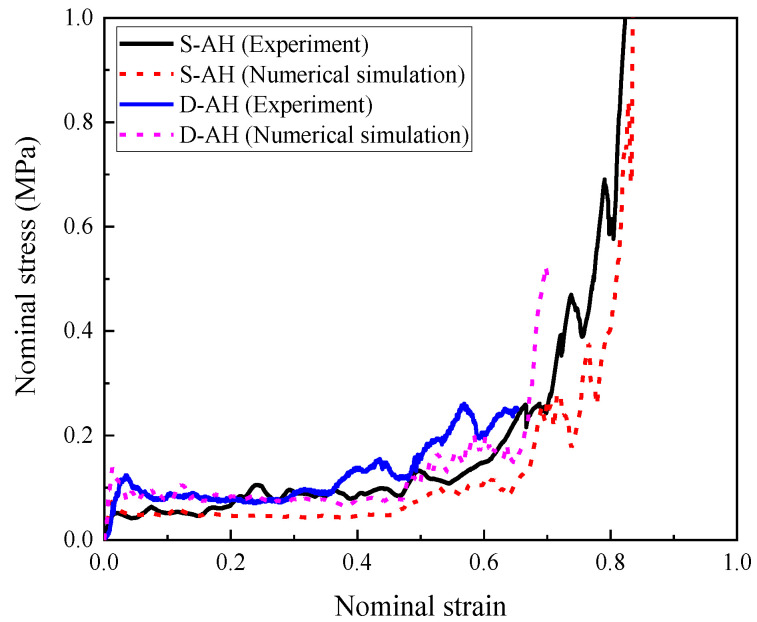
The measured and simulated stress–strain relationships of hollow auxetic honeycomb in quasi-static and dynamic compression tests.

As shown in Figure 6, the numerical model of the auxetic hollow honeycomb was calibrated with the measured stress–strain curves in the quasi-static and dynamic compression tests. The experimental data and simulation results are presented in Figure 7, and it was observed that the numerical results had a favorable agreement with the experimental data, indicating that the numerical model of the hollow honeycomb could reasonably describe its mechanical performance under quasi-static and dynamic compression. It is worth noting that the nominal stress–strain curve of the dynamic test was not to the densification stage. The reason was that an adequate buffer distance was needed for the 1 m/s crosshead to stop, otherwise, the machine would be damaged.

#### 3.2.2. Homogenous Composite

**Figure 8:**
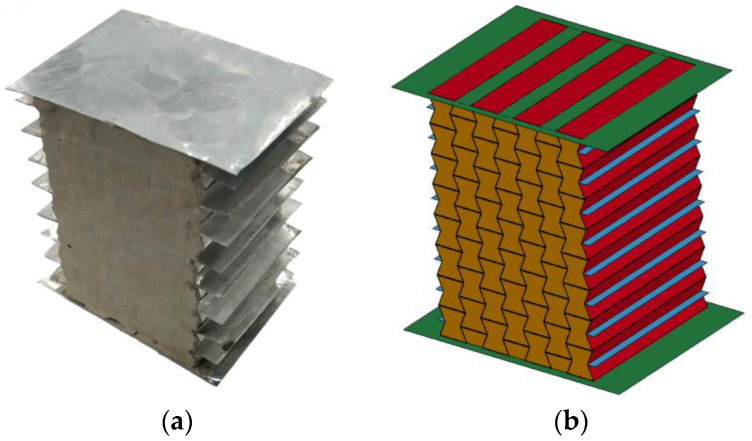
The auxetic honeycomb filled with foam concrete: (**a**) photo; (**b**) numerical model.

**Figure 9:**
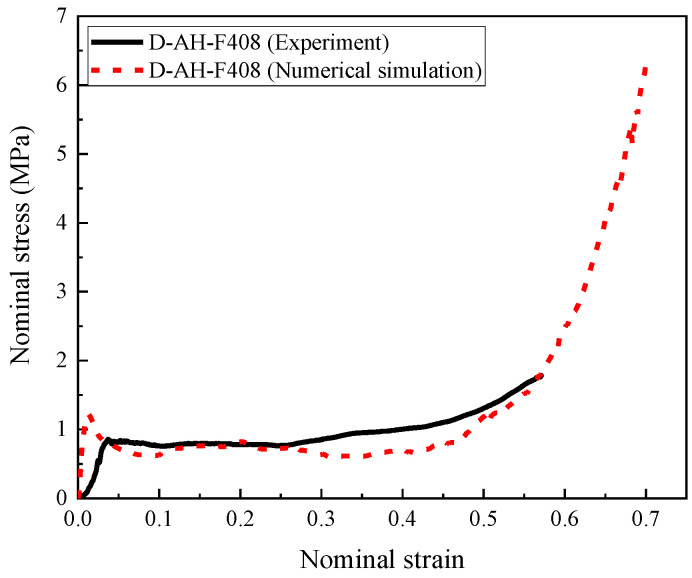
The measured and simulated stress–strain relationships of the auxetic honeycomb filled with 408 kg/m^3^ foam concrete in the dynamic compression test.

The numerical model of the aluminum honeycomb filled with 408 kg/m^3^ foam concrete was further verified by the test data, with the composite and numerical model shown in Figure 8. The experimental data and numerical simulation results are compared in Figure 9.

The results in Figure 9 show that the numerical results had a favorable agreement with the experimental results, indicating that the numerical model of the honeycomb filled with foam concrete was able to reasonably describe its mechanical performance. In addition, as shown in Figure 7 and Figure 9, it is worth noting that the peak stress and elastic modulus of the numerical results were slightly higher than the test data. The difference was mainly caused by the initial defects during the preparation process of the specimen, such as the uneven contact surfaces, incomplete filling of foam concrete, irregularity of the fabricated honeycomb, and plastic deformation of the honeycomb at the corner during the manufacturing process, etc. With increasing the compression stroke, the influence of initial defects on the mechanical performance of the auxetic hollow honeycomb and the honeycomb filled with foam concrete was moderated after the peak strain due to the appearance of more damage in the structure. For the same reason as the hollow honeycomb, the nominal stress–strain curve was not to the densification stage. In summary, the numerical model was able to reasonably simulate the dynamic response of foam concrete-filled auxetic honeycomb. However, due to the test limitation, although the numerical model was not validated in every aspect, the validated nominal stress–strain curves (or force–displacement curves) ensured the correctness and accuracy of the model to a large extent. This facilitated the further investigation of the response mode, energy absorption, and load transfer of the foam concrete-filled auxetic honeycombs with gradient.

### 3.3. Numerical Model of Graded Composite

**Figure 10:**
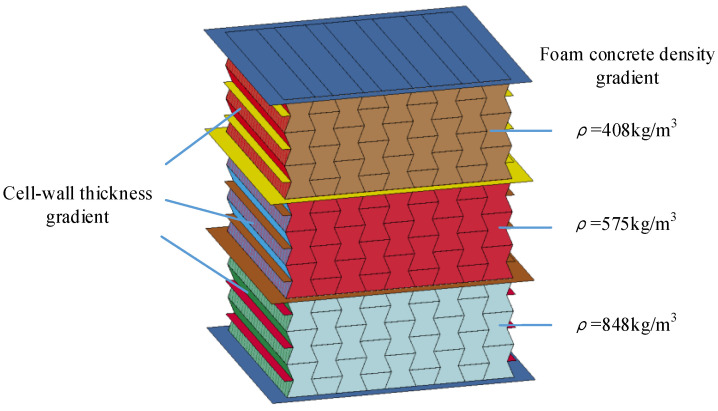
The numerical model of the composite with foam concrete density gradient: a general illustration.

From the experimental studies in Section 2, it was found that the performance of the auxetic honeycomb filled with foam concrete mainly depended on the relative strength between the honeycomb and the filled foam concrete, which was reflected by the cell-wall thickness and the filler density, respectively. From a theoretical point of view, with the same relative density, the mechanical properties and energy absorption capacity of the composite could be improved by tuning the mass distribution between the honeycomb and the foam concrete filler. Furthermore, the composite has the potential to be reasonably designed as functionally graded structures to realize multi-level structural protection with improved response mode and energy absorption characteristics. Moreover, it was known that the bearing capacity of the composite depended heavily on that of the cell wall and the foam concrete filler; meanwhile, it was convenient to tune the cell-wall thickness and the foam concrete filler density to fulfill the specified requirements. In addition, the calibrated numerical models of the hollow honeycomb and the homogenous composite in Section 3.2.1 and Section 3.2.2 provided an efficient numerical approach to investigate the response mode and energy absorption characteristics of the graded composite with consideration of the cell-wall thickness gradient and foam concrete density gradient, shown in Figure 10.

The quasi-static compressive test on the foam concrete specimen with the density of 408 kg/m^3^, 575 kg/m^3^, and 848 kg/m^3^ provided their mechanical characteristics in Figure 4b; thus, the foam concrete density gradient was determined as 408:575:848 and 848:575:408 for the positive and negative gradient designs. With the measured stress–strain curves for the dog-bone aluminum specimen, more options for the cell-wall thickness gradient were considered in the following numerical studies.

## 4. Parametric Study and Discussions

### 4.1. Influence of Cell-Wall Thickness Gradient

**Figure 11:**
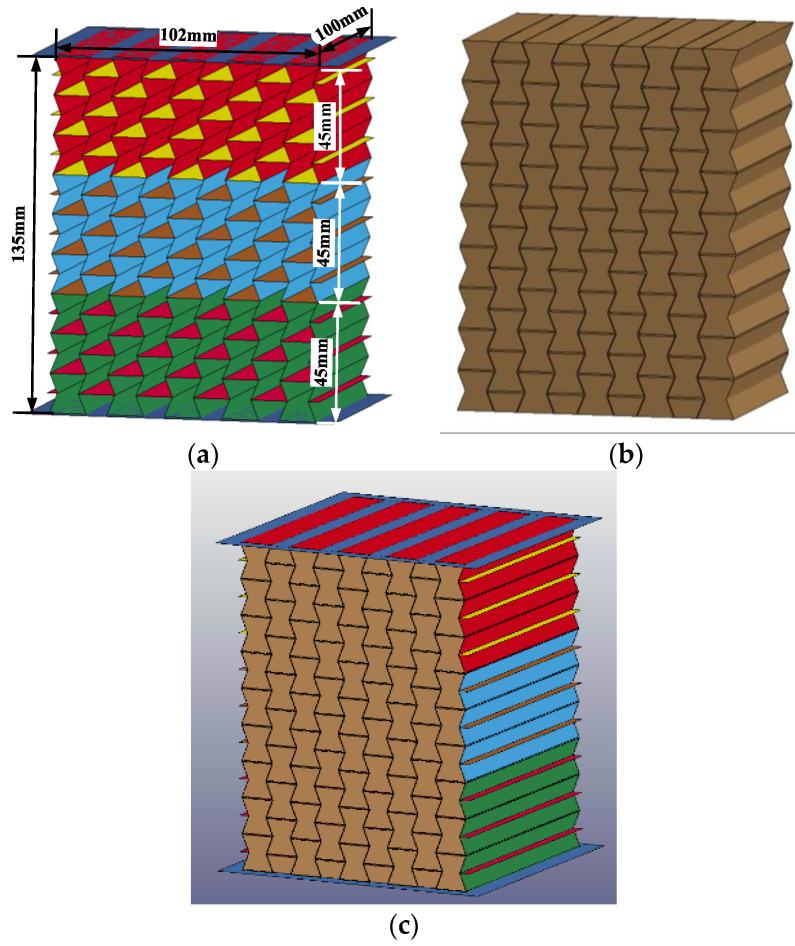
The numerical model of the composite with cell-wall thickness gradient: (**a**) auxetic honeycomb; (**b**) foam concrete filler; (**c**) auxetic honeycomb filled with foam concrete.

With the established numerical model of the graded composite, the numerical simulation was carried out to investigate the dynamic response of concave hexagonal auxetic honeycomb filled with foam concrete with layered cell-wall thickness gradient subjected to low-velocity impact, including the response mode, stress–strain characteristics, and energy absorption. Figure 11 shows the numerical models for the layered auxetic honeycomb and the foam concrete filler. The total height of the numerical model was 135 mm, while the width and the out-of-plane thickness of the core were 102 mm and 100 mm, respectively. As shown in Figure 11, the composite was divided into three layers, and the height of each layer was 45 mm. The density of foam concrete filler for all these three layers was 408 kg/m^3^, and each layer had a different thickness of cell-wall but the averaged cell-wall thickness of the graded composite remained the same as the homogenous composite.

The average thickness of the single layer of the honeycomb cell wall was set to 0.25 mm, while the thickness of the upper and lower face sheet was set to 0.5 mm, with a plane size of 118 mm × 112 mm. The total mass of the composite was 789 g and its average density was 580 kg/m^3^. In this numerical simulation, three different cell-wall thickness gradients were considered, and the cell-wall thickness of each layer was assigned as 0.2:0.25:0.3, 0.15:0.25:0.35, and 0.1:0.25:0.4, respectively. In addition, the gradient direction was also considered in this study. Table 3 lists the parameters of the seven different numerical models.

**Figure 12:**
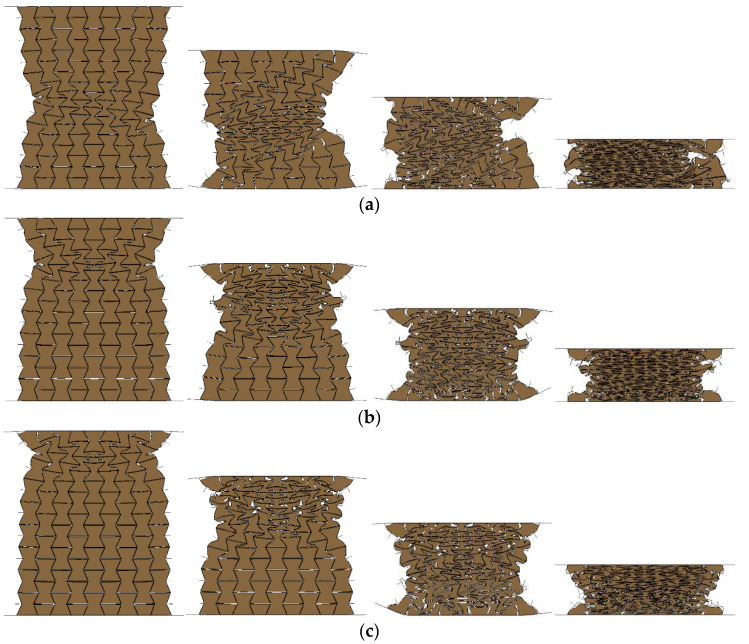

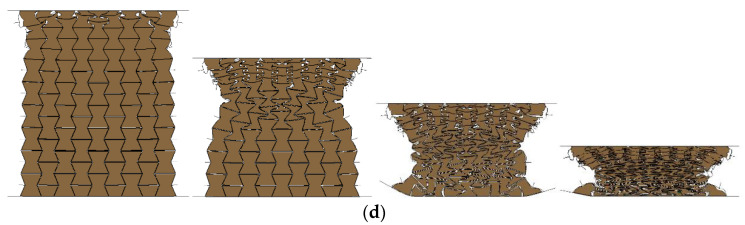
The response modes of foam concrete-filled auxetic honeycomb with different cell-wall thickness gradients at compression strokes of 15 mm, 45 mm, 75 mm, and 105 mm subjected to low-velocity impact (1 m/s): (**a**) AH-0; (**b**) PGAH-1; (**c**) PGAH-2; (**d**) PGAH-3.

Figure 12 demonstrates the response mode of the homogeneous composite and the graded composite with three positive cell-wall thickness gradients when they were subjected to the compression stroke of 15 mm, 45 mm, 75 mm, and 105 mm under the low-velocity impact of 1 m/s. As shown in Figure 12, it was found that the homogeneous composite mainly exhibited an overall response mode. The shear band first occurred in the middle of the composite, then the composite was gradually compacted with increasing compression stroke. It was noted that inadequate compaction was observed in the edge area near the upper and lower face sheet. The composite with layered cell-wall thickness tended to exhibit layered deformation at the initial stage of loading, and more obvious layered deformation was observed with relatively large cell-wall thickness gradients. The homogeneous composite and graded composite with different cell-wall thickness gradients both exhibited the negative Poisson’s ratio effect, reflected by the concave deformation of the composite. Meanwhile, compared with the negative Poisson’s ratio effect in these three layers with different cell-wall thicknesses, it was also observed that the concave deformation was more obvious in the layer with the thickest cell-wall due to the sufficient constrain effect applied on the foam concrete filler. As shown in Figure 12, the composite exhibited more obvious concave deformation from top to bottom with a larger positive cell-wall thickness gradient. Thus it is concluded that the relatively thin cell wall is unable to effectively constrain the foam concrete deformation and produce the expected concave deformation mode, leading to an unobvious negative Poisson’s ratio effect. In summary, the appropriate arrangement of the cell-wall thickness gradient has the potential to realize the layered response at the initial loading stage and the negative Poisson’s ratio effect with sufficient constraint provided by cell-wall with a certain thickness.

**Figure 13:**
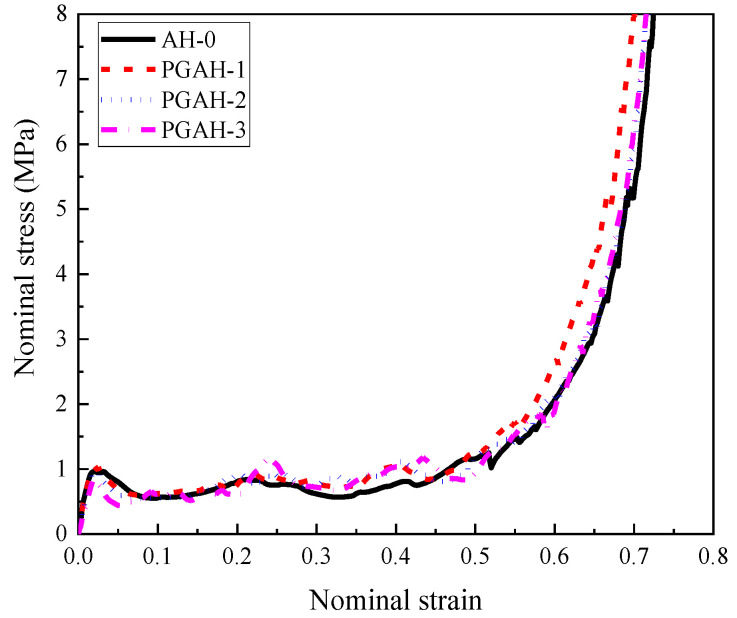
The comparison of the stress–strain relationships of the homogenous composite and graded composite with different cell-wall thickness gradients subjected to low-velocity impact (1 m/s).

The stress–strain relationship of the composite with different cell-wall thickness gradients was compared in Figure 13, suggesting that the cell-wall thickness gradient had a negligible influence on the load-bearing capacity of the composites. Furthermore, although the homogenous and graded composite both exhibited a relatively steady stress plateau in the stress–strain curve, it was also observed that the homogeneous composite AH-0 provided a more steady stress plateau, and the stress plateau was more fluctuated with increasing cell-wall thickness gradient. The graded composite demonstrated a trend of progressive collapse-type compression deformation due to the different cell-wall thicknesses for different layers, leading to different bearing capacities in the stress plateau provided by different layers. The layer with a relatively thick cell wall provided relatively higher plateau stress compared with those with relatively thin cell walls, leading to slightly fluctuated and gradually increased stress plateau corresponding to their compression deformation mode. When the top layer with the thinnest cell wall started to compress, the middle and bottom layers remained in the elastic stage. Thus the first peak stress reflected the load-bearing capacity of the top layer. With increasing compression stroke, the crushing would occur in the middle layer when its peak stress was reached, and the same phenomenon would happen in the bottom layer. Therefore, from the stress–strain relationships of the composites with cell-wall thickness gradients, three peak stresses were observed, indicating the load-bearing capacity for these respective three layers. 

**Figure 14:**
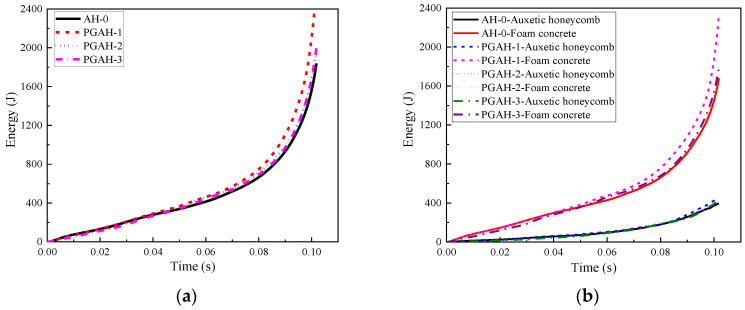
The time history of absorbed energy (1 m/s): (**a**) foam concrete filled auxetic honeycombs; (**b**) foam concrete and auxetic honeycomb.

In addition to the mechanical characteristics of the homogenous or graded composites with different cell-wall thickness gradients in Figure 13, some other parameters for each component in the composite could be obtained with the keywords *DATABASE_MATSUM, such as the kinetic energy, internal energy, etc. Figure 14a demonstrates the time history of energy absorbed by the composites with different cell-wall thickness gradients, implying that the cell-wall thickness gradient had a limited influence on the energy absorption of the composite. During the initial loading stage, the compression deformation mainly occurred in the layer with the thinnest cell wall; thus, PGAH-3 exhibited slightly less energy absorption capacity compared with other graded composites when the compression stroke was less than 40 mm. Moreover, the homogeneous composite AH-0 presented an overall deformation, but the two side cells near the top and bottom face sheets were insufficiently compacted, leading to slightly weaker energy absorption than the graded composite during the later stage of compression. The results show that the cell-wall thickness gradient had a significant effect on the deformation mode of the composites but a limited influence on their mechanical performance and energy absorption capacity. Therefore, with the same overall average density of the aluminum cell wall and foam concrete filler, the appropriate arrangement of the cell-wall thickness gradient of the composite was beneficial to realizing the ideal layer-by-layer compression deformation mode, which had great potential to be designed as a layered functionally graded sacrificial cladding for multi-level protection.

Figure 14b shows the energy absorbed by the foam concrete filler and auxetic honeycomb, respectively, indicating that the energy absorption capability of the composite depended mainly on the foam concrete filler. As shown in Figure 14b, the absorbed energy by the foam concrete filler and auxetic honeycomb linearly increased with increasing compression stroke, then exhibited a rapid increase during the later loading stage. In general, the composite with the cell-wall thickness gradient showed a lower compressive strength (comparatively lower initial peak stress, shown in Figure 13), and higher energy absorption in Figure 14a, which was beneficial to energy absorption improvement as well as initial impact mitigation if the composite was designed as the sacrificial cladding to protect important structural components against impact load.

**Figure 15:**
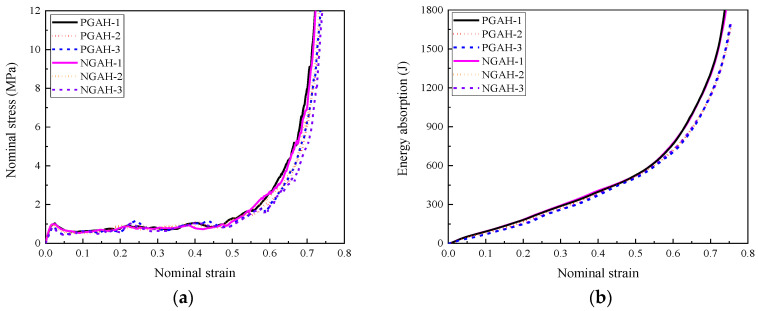
The stress–strain relationships and energy absorption of the composite with different cell-wall thickness gradients and gradient directions (1 m/s): (**a**) stress–strain relationships; (**b**) energy absorption.

The stress–strain curves and energy absorption performance of the composites with different cell-wall thickness gradient directions are shown in Figure 15. It was found that the cell-wall thickness gradient direction had a limited effect on the load-bearing capacity under a relatively low loading rate (1 m/s in the present study). In addition, the total energy absorbed by the composite was almost the same, implying that the effect of gradient direction on their energy absorption performance was negligible, shown in Figure 15b.

Based on the results and discussions above, it was concluded that a reasonable arrangement of the cell-wall thickness gradient of the auxetic honeycombs filled with foam concrete was beneficial to achieving the expected staged response mode, providing an alternative approach for specific multi-level protection simply and conveniently.

### 4.2. Influence of Foam Concrete Density Gradient

**Figure 16:**
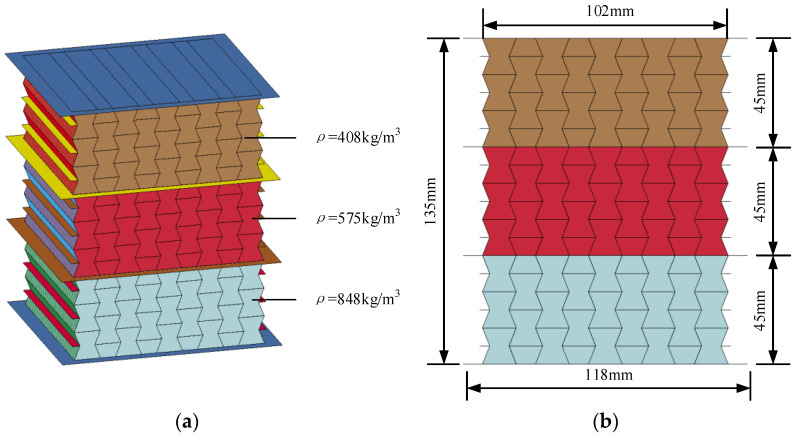
The numerical model and geometric parameters of the composite with foam concrete density gradient (1 m/s): (**a**) numerical model; (**b**) geometric parameters.

As shown in Figure 16, the graded composite with foam concrete density gradient was considered, in which the foam concrete density was assigned as 408 kg/m^3^, 575 kg/m^3^, and 848 kg/m^3^ for the top, middle, and bottom layers, respectively. To investigate the effect of foam concrete density gradient on the response mode, load transfer, and energy absorption capacity of the composite, the thickness of the cell wall of these three layers was set identical. Moreover, two separation plates were introduced between different layers, whose geometries were the same as those of the face plates. 

**Figure 17:**
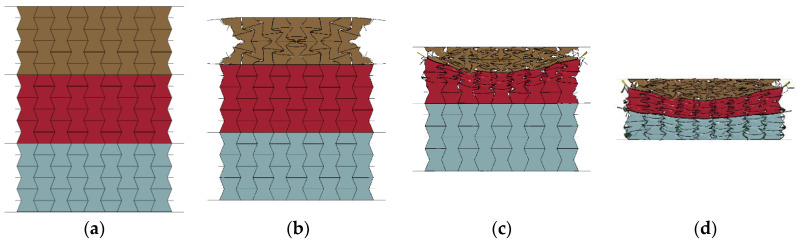
The response modes of foam concrete-filled auxetic honeycombs (*t* = 0.2:0.2:0.2 and *ρ* = 408:575:848) at different compression strokes (1 m/s): (**a**) *D* = 0 mm; (**b**) *D* = 15 mm; (**c**) *D* = 55 mm; (**d**) *D* = 95 mm.

Figure 17 presents the response mode of the composite with a cell-wall thickness of 0.2 mm for all three layers subjected to compression strokes of 0, 15, 55, and 95 mm, respectively. As shown in Figure 17, the composite exhibited a significant staged response mode, and these three layers were compressed and compacted in sequence from the top layer to the bottom layer. During the initial stage of compression loading, the 0.2 mm thick cell wall was able to effectively constrain the 408 kg/m^3^ foam concrete filler, resulting in an obvious negative Poisson’s ratio effect in the top layer, as shown in Figure 17b. When the compression stroke reached 55 mm, uneven compression loading was transferred to the middle layer due to the material concentration in the center of the top layer by its significant negative Poisson’s ratio effect, as shown in Figure 17c. The bottom layer also had to endure uneven compression due to the sagged deformation in the center of the middle layer, when the compression stroke increased to 95 mm, as shown in Figure 17d. Due to the larger compression load in the center transferred to the middle and bottom layers, these two layers were more difficult to realize the expected negative Poisson’s ratio effect. Despite this, the lateral deformation of the foam concrete filler was significantly mitigated by the aluminum honeycomb, as shown in Figure 17c,d. The composite showed an obvious layer-by-layer compression response mode but only the top layer exhibited a significant negative Poisson’s ratio effect.

**Figure 18:**
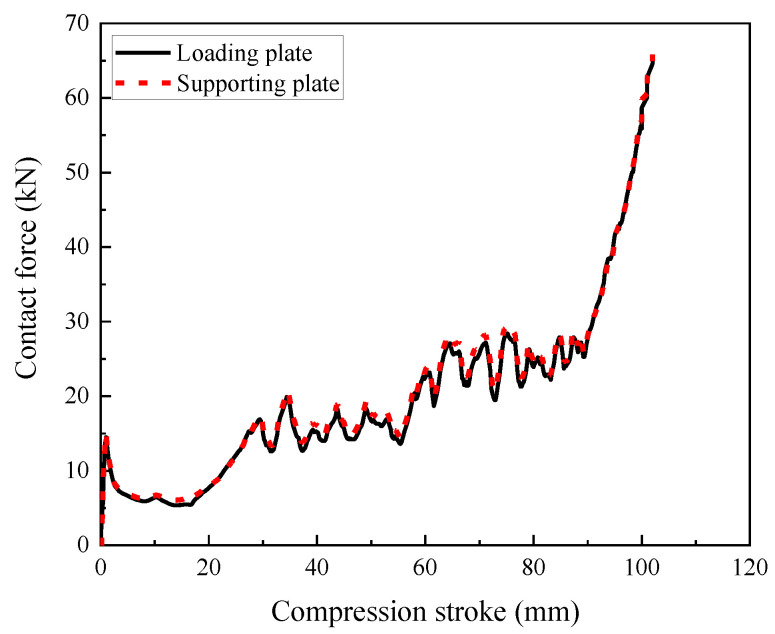
The contact forces between the composite and the loading or supporting plates (*t* = 0.2:0.2:0.2 and *ρ* = 408:575:848, 1 m/s).

The keyword *DATABASE_RCFORC output was adopted to obtain the contact force between the composite and the upper loading or lower supporting plates in Figure 18, which was applied to evaluate the load-carrying capacity. It was worth noting that the contact force between the composite and the supporting plate was also regarded as the load transferred to the protected structure, which was important data to evaluate the protection performance of the composite when it was applied as the sacrificial cladding. As shown in Figure 18, the composite with foam concrete density gradient presented three increasing plateau stresses with increasing compression stroke, corresponding to the compression deformation process of these three layers in Figure 17. It was also observed that the top layer exhibited higher initial peak stress and a more steady stress plateau mainly due to its obvious negative Poisson’s ratio effect. Differently, the middle and bottom layers demonstrated inconspicuous peak stress and fluctuated stress plateau, since the foam concrete filler in these two layers was difficult to provide steady and continuous support by the inconspicuous negative Poisson’s ratio effect. Meanwhile, the continuous crushing of cells in the middle and bottom layers would affect the loading-bearing capacity of the composite, resulting in a fluctuated stress plateau. In addition, the bearing capacity of the composite demonstrated a rapid increase after the complete densification of these three layers.

The plateau stress, densification strain, and effective energy absorption of the composite at different compression stages are listed in Table 4. The compression stage was simply determined according to the platform of the load–displacement curve in Figure 18, and the peak stress was taken as the dividing points of different compression stages in the present study. As shown in Figure 18, the nominal strain ranges of 0–0.216, 0.216–0.45, and 0.45–0.662 corresponded to the first, second, and third compression stages, respectively. Meanwhile, the observed response mode of the composite in Figure 17 shows that major deformation in the first, second, and third stages focused on the top, middle, and bottom layers of the structure, respectively. Prior to densification, the total energy absorption of the structure was 1474.39 J, while the absorbed energy at the first, second, and third stages was 243.41, 520.94, and 710.04 J, accounting for 16.51%, 35.33%, and 48.16% of the total energy absorption, respectively. It showed that the total energy absorbed at each compression stage increased with increasing foam concrete density. In summary, it was concluded that the appropriate arrangement of the foam concrete density gradient was able to realize the sequential compression response for multi-level protection. 

**Figure 19:**
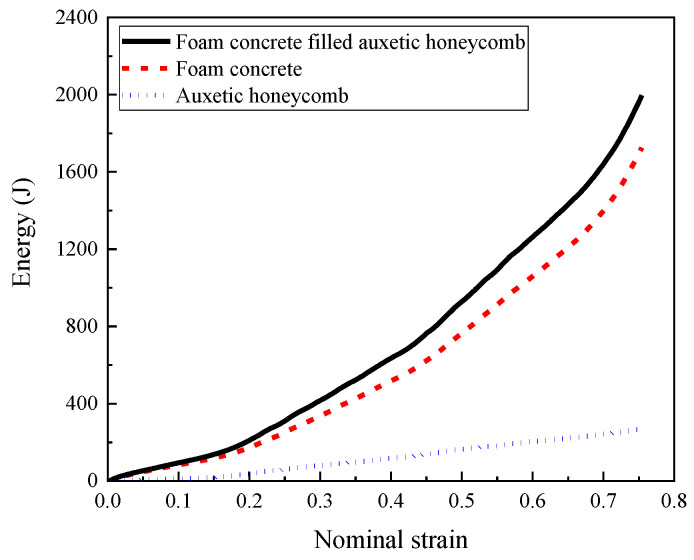
The energy absorption performance of the auxetic honeycomb, foam concrete, and composite (*t* = 0.2:0.2:0.2 and *ρ* = 408:575:848, 1 m/s).

The absorbed energy by the auxetic honeycomb, foam concrete filler, and the composite is shown in Figure 19, suggesting that the foam concrete filler contributed to the majority of the energy absorption of the composite. Although the auxetic honeycomb exhibited insignificant energy absorption performance, it provided sufficient constraint for the foam concrete filler, which could effectively mitigate the brittle fracture failure of the foam concrete to significantly improve the energy absorption of the foam concrete filler. 

**Figure 20:**
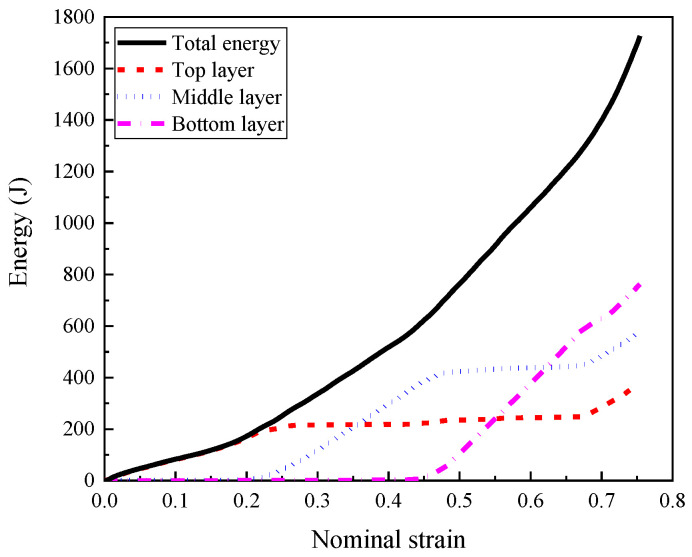
The comparison of energy absorption of layered foam concrete during compression (*t* = 0.2:0.2:0.2 and *ρ* = 408:575:848, 1 m/s).

The energy absorption performance of the foam concrete filler in each layer with respect to the nominal strain is shown in Figure 20, and the details were listed in Table 5. Before the complete densification of the composite, the total energy absorbed by the foam concrete filler was 1248.35 J, which was 198.39 J, 425.37 J, and 624.59 J in each compression stage, accounting for 15.89%, 34.07%, and 50.04% of the total energy absorption. The huge difference in the absorbed energy among these three layers was mainly due to the remarkably different plateau stress of these three layers filled with different densities of foam concrete. Furthermore, the energy absorbed by the top layer of foam concrete accounted for 96.42% of the total absorbed energy by the composite during the first compression stage. During the second compression stage, the majority of deformation and energy absorption occurred in the middle layer, while the top and bottom layers only contributed to 11.28% of the total absorbed energy. In this compression stage, it was noted that the top layer entered the densification stage, leading to continuous energy absorption. In the third compression stage, the bottom layer absorbed 86.94% of the total energy, meanwhile, the top and middle layers reached the densification stage and contributed 13.06% of the total energy. In summary, from Figure 20 and Table 5, the staged compression response mode of the composite with foam concrete density gradient can realize layer-by-layer energy absorption due to the sufficient strength difference among these three layers.

In summary, it was found that the graded composite with foam concrete density gradient exhibited superior performance to the homogenous composite from the viewpoint of structural protection. It is known that the composite filled with high-density foam concrete provided high plateau stress, resulting in strong energy absorption. However, it also brings high initial peak stress for the protected structure, which is unfavorable for structure protection. The proposed graded composites with foam concrete density gradient could realize low initial peak stress and long stress plateau, implying that it may effectively mitigate the load transfer to the protected structures and reduce the potential damage. In addition, the sequential compression response mode could achieve the purpose of multi-level protection, for instance, only the damaged outer layer is required to be replaced after a small collision in practice.

**Figure 21:**
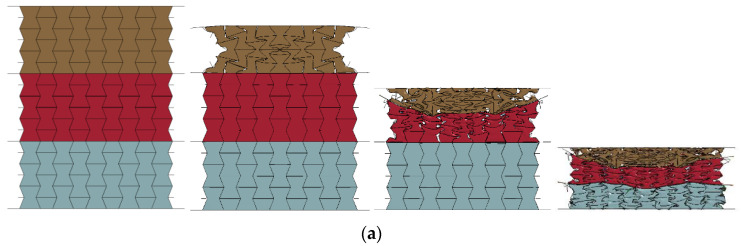

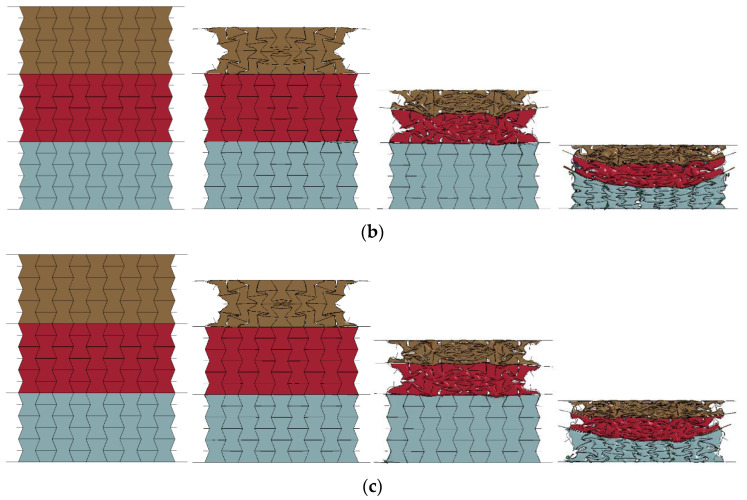
The response modes of the composites with foam concrete density gradient under compression strokes of 0, 15, 55, and 95 mm (1 m/s): (**a**) *t* = 0.3:0.3:0.3 and *ρ* = 408:575:848; (**b**) *t* = 0.4:0.4:0.4 and *ρ* = 408:575:848; (**c**) *t* = 0.5:0.5:0.5 and *ρ* = 408:575:848.

The previous results in Figure 17, Figure 18, Figure 19 and Figure 20 illustrate the performance of the composite with a 0.2 mm thick cell wall and filled with three different densities of foam concrete, namely 408 kg/m^3^, 575 kg/m^3^, and 848 kg/m^3^, respectively. From the response mode in Figure 17, it was observed that only the top layer exhibited a favorable negative Poisson’s ratio effect. To investigate the effect of cell-wall thickness on the performance of the composite with foam concrete density gradient, three more different cell-wall thicknesses, namely, 0.3 mm, 0.4 mm, and 0.5 mm were considered herein, and the corresponding response modes are shown in Figure 21. It was found that increasing the thickness of cell-wall was able to further constrain the lateral expansion of the foam concrete filler, which was beneficial for achieving the favorable negative Poisson’s ratio effect for all these three layers. Thus when the cell-wall thickness increased to 0.5 mm, the top and middle layers both exhibited obvious concave deformation, and the thick cell wall could restrain the lateral expansion of the bottom layer to a great extent.

**Figure 22:**
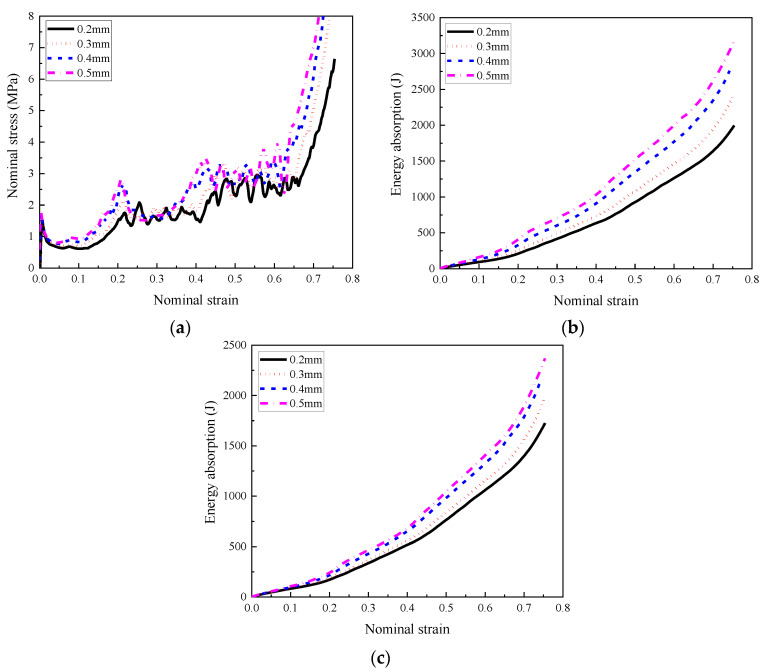
The stress–strain relationships and energy absorption of the composite (*ρ* = 408:575:848) with different cell-wall thicknesses (1 m/s): (**a**) stress–strain relationships; (**b**) energy absorption of the composite; (**c**) energy absorption of the foam concrete.

The stress–strain relationships of the composites with foam concrete density gradient and different thicknesses of cell-wall are shown in Figure 22a, and it was found that the initial peak stress and plateau stress increased with increasing cell-wall thickness, implying that thicker cell wall imposed stronger constrain to the foam concrete filler. For composites with these four different cell-wall thicknesses, the top layer demonstrated an obvious negative Poisson’s ratio effect; thus, obvious initial peak stress and steady stress plateau were observed during the first compression stage. During the second compression stage, since the 0.2 mm thick cell wall was difficult to fully constrain the lateral expansion of foam concrete in the middle layer, resulting in the fluctuated stress plateau. By the presented compression deformation mode in Figure 21, compared with the 0.2 mm thick cell wall, the 0.3 mm thick cell wall imposed stronger constrain on the foam concrete in the middle layer despite its inconspicuous negative Poisson’s ratio effect, leading to slightly fluctuated stress plateau. The composites with cell-wall thicknesses 0.4 mm and 0.5 mm exhibited obvious negative Poisson’s ratio effect, resulting in high peak stress and steady stress plateau during the second compression stage. When it entered the third compression stage, all the composites with cell-wall thicknesses of 0.2 mm, 0.3 mm, 0.4 mm, and 0.5 mm exhibited insignificant negative Poisson’s ratio effect, resulting in fluctuated stress plateau.

With cell-wall thicknesses of 0.2 mm, 0.3 mm, 0.4 mm, and 0.5 mm, Figure 22b,c demonstrated the energy absorption of the composites and the foam concrete filler, respectively. These results showed that the energy absorption of the composite and the foam concrete filler increased with increasing cell-wall thickness. It implied that increasing the cell-wall thickness was able to improve the constraining effect on the foam concrete filler, contributing to higher peak stress and plateau stress during these three compression stages, as well as higher energy absorption. Although the composite with thicker cell walls exhibited relatively higher stress, it suffered from the comparatively smaller densification strain, which was unfavorable for total energy absorption. In addition, the quickly increased peak stress and plateau stress were also unfriendly to the protected structures, implying that larger loads would be transferred to the protected structures. On one hand, from the theoretical point of view, the ideal cell-wall thickness is mainly determined by the factors of realizing the staged compression response mode, reducing the peak stress, and improving the total energy absorption, when the proposed composite is applied as the sacrificial cladding to protect important structural components. On the other hand, from the practical point of view, it is necessary to consider the specific requirement of the protected structures. For instance, to achieve multi-level protection, the foam concrete density and cell-wall thickness in each layer should be determined by their requirement of absorbed energy to resist small, medium, or large collisions.

### 4.3. Influence of Loading Rate

**Figure 23:**
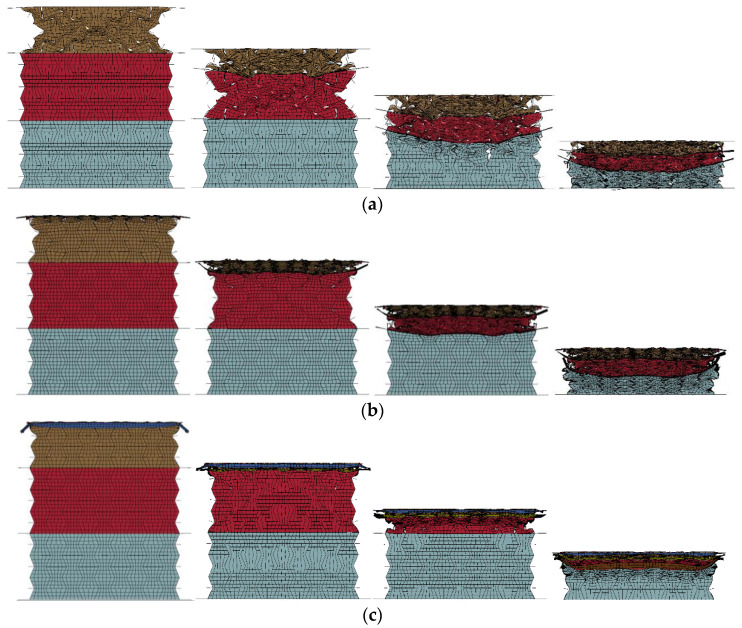
The response modes of the composite (*t* = 0.4:0.4:0.4 and *ρ* = 408:575:848): (**a**) quasi-static mode (1 m/s); (**b**) transitional mode (10 m/s); (**c**) progressive collapse mode (100 m/s).

Cellular materials and structures exhibited different response modes and energy absorption at different compression rates. Therefore, different impact velocities, namely, 1 m/s, 2 m/s, 5 m/s, 10 m/s, 20 m/s, 50 m/s, and 100 m/s were applied to the graded composites to investigate the failure mode, load transfer, and energy absorption characteristics. Due to its comparatively superior performance in the aspects of its deformation mode, load transfer, and energy absorption compared with other composites, the composite with cell-wall thickness of 0.4 mm and filled with 408, 575, and 848 kg/m^3^ foam concrete in its top, middle, and the bottom layer was chosen to investigate the influence of the loading rate herein. The deformation mode of the composite under low (1 m/s), medium (10 m/s), and high compression velocities (100 m/s) were illustrated in Figure 23, in which different compressive response modes were observed. From Figure 23a, with a low loading rate, the composite underwent layered compression mode and the top and middle layers exhibited a significant negative Poisson’s ratio effect. Subjected to a medium loading rate, the composite also demonstrated a remarkably layered compression but a limited negative Poisson’s ratio effect. With a high loading rate, the composite underwent progressive collapse from the loading end throughout the entire response process.

**Figure 24:**
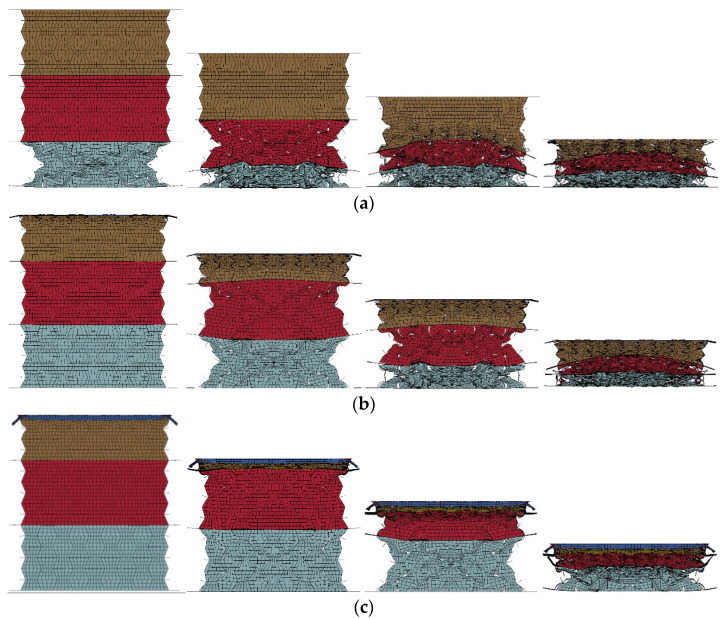
The response modes of the composite (*t* = 0.4:0.4:0.4 and *ρ* = 848:575:408): (**a**) quasi-static mode (1 m/s); (**b**) transitional mode (10 m/s); (**c**) progressive collapse mode (100 m/s).

Furthermore, the deformation mode of the composite with negative foam concrete density gradient is shown in Figure 24, and it was found that the loading rate had a significant effect on the deformation mode. Filled with foam concrete with density 848, 575, and 408 kg/m^3^ from top to bottom layer, the composite exhibited layered deformation mode due to the different strengths of these layers under quasi-static loading. In contrast, with medium loading rates, all these three layers started to deform almost simultaneously while the composite still showed layered compression deformation. With a high loading rate, the composite underwent progressive collapse, while the deformation initiated and propagated with the loading plate, exhibiting top-to-bottom deformation mode despite the negative foam concrete density gradient arrangement. In summary, it was found that the response mode of the composite with foam concrete density gradient was significantly affected by the loading rate. Under low loading rates, the deformation sequence of each layer was determined by its strength. With medium loading rates, the strength of these layers and the loading speed both influenced the deformation sequence of each layer. With high loading rates, it was observed that the deformation sequence was mainly affected by the loading speed.

**Figure 25:**
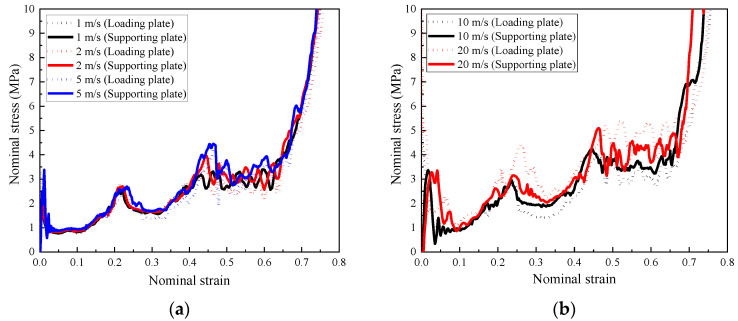
The stress–strain relationships of the composite calculated from the contact forces between the composite and loading plate and supporting plate (*t* = 0.4:0.4:0.4 and *ρ* = 848:575:408): (**a**) 1 m/s, 2 m/s, and 5 m/s; (**b**) 10 m/s and 20 m/s.

Subjected to high loading speeds, such as 50 m/s and 100 m/s, it was difficult to avoid the high initial peak stress, which was unfavorable for structural protection. In practice, the impact velocity of vehicles is lower than 50 m/s in most collision accidents. Therefore, in the present study, low and medium loading rates were considered to investigate the performance of the composite with foam concrete density gradient, shown in Figure 25. The difference between the two contact forces at the two ends of the composite increased with increasing loading rate, which could be applied to evaluate the impact load mitigation for the protected structure. Meanwhile, it was observed that the peak stress had a significant increase with increasing loading rate, as shown in Figure 25b. Differently, the influence of loading rate on the plateau stress was unremarkable. One of the typical applications of the proposed composite is sacrificial cladding to protect important structural components, it is more concerned about the load transferred to the protected structural components, and it was observed that the composite can significantly reduce the load transferred to the protected structure, especially under high impact velocities.

**Figure 26:**
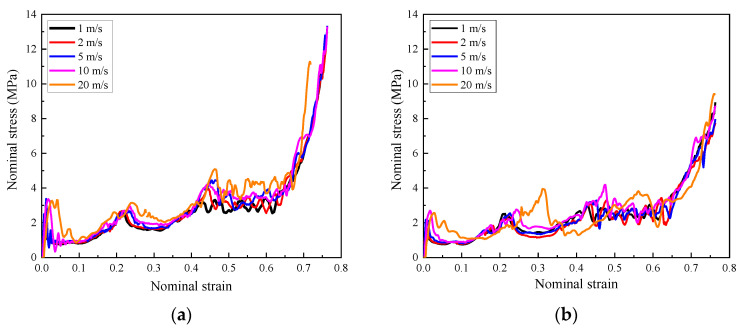
The stress–strain relationships of the composite (*t* = 0.4:0.4:0.4) with different foam concrete density gradient directions: (**a**) positive gradient (*ρ* = 408:575:848); (**b**) negative gradient (*ρ* = 848:575:408).

Figure 26 shows the influence of the foam concrete density gradient direction on the nominal stress of the composite under different loading rates, which showed that they provided a comparable bearing capacity except for the small difference that the initial peak stress of the composite with positive foam concrete density gradient was 21% higher than that with negative foam concrete density gradient at low and medium loading rates.

**Figure 27:**
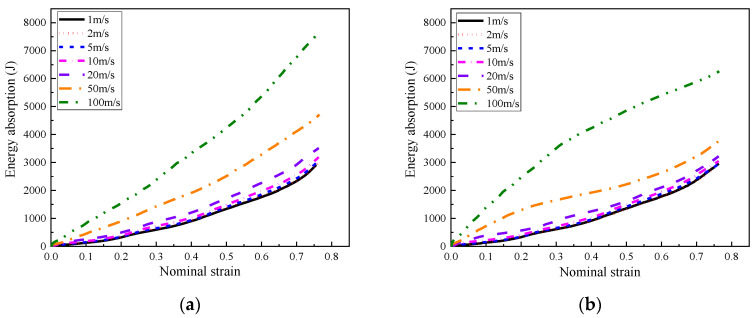
The energy absorption of the composite (*t* = 0.4:0.4:0.4) with different foam concrete density gradient directions: (**a**) positive gradient (*ρ* = 408:575:848); (**b**) negative gradient (*ρ* = 848:575:408).

The energy absorption capacity of the composite with positive and negative foam concrete density gradients under different loading rates is also presented in Figure 27. The results demonstrated that the influence of the dynamic effect on the energy absorption capacity of the composite was limited for the loading rate of lower than 20 m/s. In contrast, with loading rates of 50 m/s or 100 m/s, the energy absorption significantly increased with increasing loading rate.

**Figure 28:**
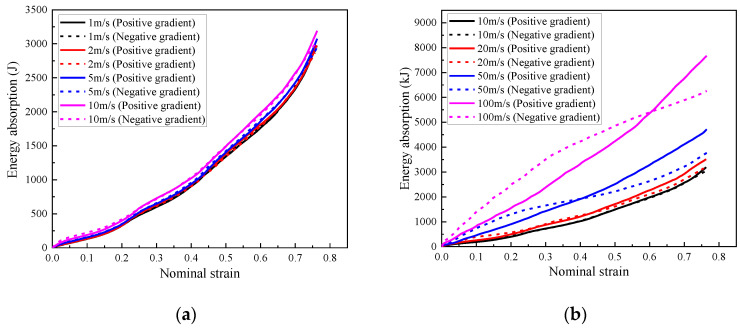
The energy absorption of the composite (*t* = 0.4:0.4:0.4) with positive gradient (*ρ* = 408:575:848) or negative gradinet (*ρ* = 848:575:408) under different loading rates: (**a**) 1 m/s, 2 m/s, 5 m/s and 10 m/s; (**b**) 10 m/s, 20 m/s, 50 m/s and 100 m/s.

As shown in Figure 28a, under low and medium loading rates, the composite with positive or negative foam concrete density gradient demonstrated almost the same energy absorption capacity due to their identical layered compression response mode, starting from the layer with the lowest foam concrete density and propagating to the layer with the highest foam concrete density. In contrast, when with high loading rates, since the compression deformation initiated in the layer close to the loading plate and developed to the supporting plate, the composite with positive or negative foam concrete density gradient exhibited different energy absorption capacity, as shown in Figure 28b. In the composite with a positive foam concrete density gradient, the layered compression started in the layer filled with the lowest foam concrete density and ended in the layer with the highest foam concrete density. However, the composite with a negative foam concrete density gradient showed a reversed layered compression response sequence. Therefore, as shown in Figure 28b, the composite with positive foam concrete density gradient showed increasing energy absorption efficiency, judged by the growing trend of the energy absorption, but the composite with negative foam concrete density gradient exhibited first higher and then lower energy absorption efficiency with increasing nominal strain. In summary, the composite with positive or negative foam concrete density gradient presented almost the same energy absorption under low and medium loading rates. Under high loading rates, the composite with positive foam concrete density gradient exhibited lower energy absorption at the initial stage of compression but higher energy absorption at the late stage of compression, compared with the composite with negative foam concrete density gradient.

### 4.4. Effective Poisson’s Ratio

**Figure 29:**
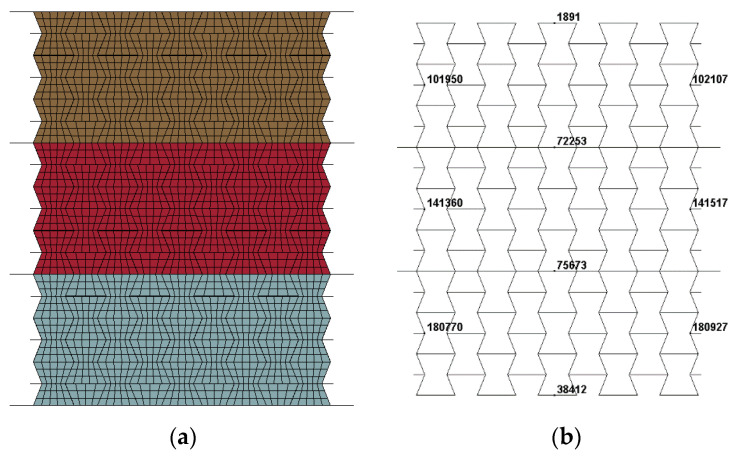
The representative points for effective Poisson’s ratio calculation: (**a**) numerical model; (**b**) representative points.

Compared with positive Poisson’s ratio structures, negative Poisson’s ratio structures demonstrated excellent performance in the aspects of stress distribution, shear modulus, indentation resistance, impact resistance, and energy absorption [51]. In the present study, the effective Poisson’s ratio [63] was employed to investigate the effect of loading rate on the Poisson’s ratio of each layer. As shown in Figure 29, some representative points in the numerical model were selected to evaluate the effective Poisson’s ratio of each layer. Based on the horizontal and vertical displacement of these points, the relative displacement between the corresponding points of each layer could be calculated to determine the effective Poisson’s ratio of each layer in the composite. As shown in Figure 29b, the effective Poisson’s ratio could be evaluated by the horizontal nominal strain divided by the vertical nominal strain. The former was obtained by the relative horizontal displacement divided by the initial distance of the two points at two sides of each layer, while the latter was obtained by the relative vertical displacement divided by the initial distance of the two points at the top and bottom of each layer.

**Figure 30:**
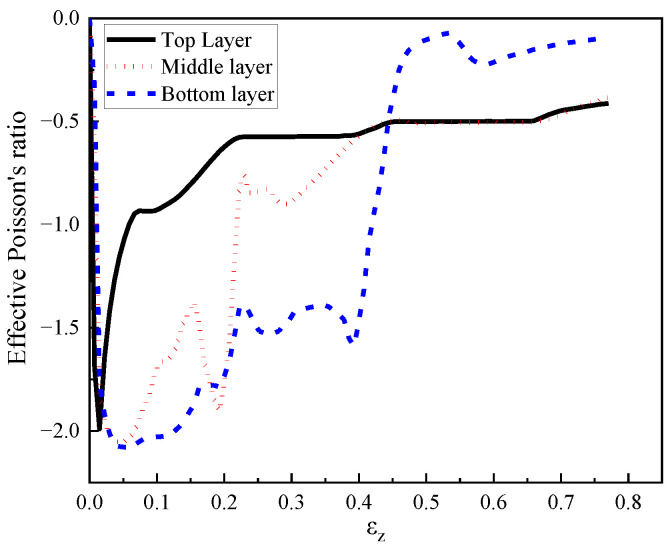
The effective Poisson’s ratio of each layer at a loading rate of 2 m/s (*t* = 0.4:0.4:0.4 and *ρ* = 408:575:848).

The composite with cell-wall thickness of 0.4 mm as well as filled with 408 kg/m^3^, 575 kg/m^3^, and 848 kg/m^3^ foam concrete from the top to the bottom layer, was adopted herein to investigate the effective Poisson’s ratio of these three layers. Figure 30 demonstrates the effective Poisson’s ratio of each layer under the loading rate of 2 m/s. Due to small compression deformation during the initial loading stage, the honeycomb could effectively constrain the lateral expansion of the foam concrete filler in each layer, resulting in the expected concave response mode. With increasing compression stroke, the compaction of foam concrete continued, but the absolute value of effective Poisson’s ratio gradually decreased, as shown in Figure 30. It was known that the staged compression response initiated in the top layer; thus, its absolute value of effective Poisson’s ratio had a rapid decrease with increasing vertical strain. After the densification of the top layer, the middle layer started to deform and its effective Poisson’s ratio also underwent a significant decrease. Similarly, the bottom layer exhibited a sharp decrease in the absolute value of effective Poisson’s ratio after the densification of the middle layer.

**Figure 31:**
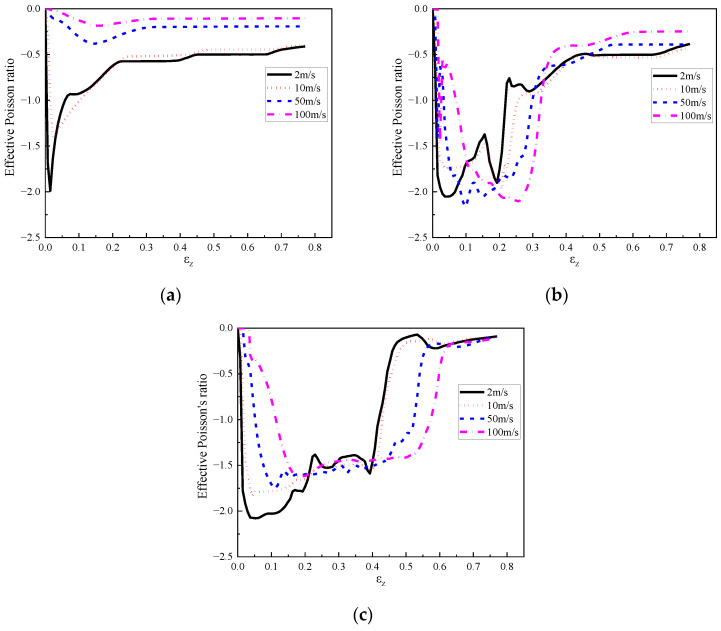
The effective Poisson’s ratio of the composite (*t* = 0.4:0.4:0.4 and *ρ* = 408:575:848) at loading rates of 2 m/s, 10 m/s, 50 m/s, and 100 m/s: (**a**) top layer; (**b**) middle layer; (**c**) bottom layer.

Figure 31 demonstrates the effective Poisson’s ratio of each layer under different loading rates. It was observed that with loading rates of 2 m/s and 10 m/s, the top layer exhibited an obvious negative Poisson’s effect at the end of loading, while the negative Poisson’s effect in the top layer was insignificant under loading rates of 50 m/s and 100 m/s where the crushing failure occurred. A similar phenomenon was also observed in the middle layer, increasing the loading rate would decrease the negative Poisson’s effect at the final stage. Due to the uneven initial deformation under low and medium loading rates as well as the progressive collapse under high loading rates, the bottom layer exhibited insignificant negative Poisson’s effect at the end of loading. In summary, it was concluded that the three layers in the composite with foam concrete density gradient exhibited an obvious negative Poisson’s ratio at the initial loading stage due to the concave design of hexagon cells, while the negative Poisson’s ratio effect gradually attenuated with increasing compression stroke as the foam concrete filler was gradually compacted. In addition, the negative Poisson’s ratio effect of each layer decreased with increasing loading rate.

## 5. Conclusions

In the present study, a novel graded auxetic foam concrete-filled honeycomb was proposed to exhibit layered response with staged load transfer and energy absorption, which was promising to be applied as sacrificial cladding to realize multi-level protection for important structural components. In the proposed layered composite, the strength gradient of different layers was realized by tuning the thickness gradient of honeycomb cell-wall and density gradient of foam concrete filler, whose effect on the deformation mode, initial peak stress, plateau stress, energy absorption, and effective Poisson’s ratio was systematically investigated with a validated numerical model. The main conclusions were drawn as follows,

(1) The composite with cell-wall thickness gradient exhibited layered response mode under low or medium loading rates (1 m/s, and 10 m/s, respectively). Increasing the cell-wall thickness gradient led to a more obvious layer-by-layer compression response, lower initial peak stress, and a more fluctuated stress plateau. Compared with the homogenous cell-wall thickness counterpart, the improvement of the energy absorption of the composite with cell-wall thickness gradient was minor, less than 5%.
(2) The foam concrete density gradient and direction had a significant effect on the performance of the composite, reflected by the observed three-stage stress plateau corresponding to the layered compression. In the first, second, and third deformation stages, the top layer, middle layer and bottom layer foam concrete of the composite with positive gradient absorbed 94.62%, 88.72%, and 86.94% of the total foam concrete energy absorption, respectively. The layered architecture filled with foam concrete of different densities was able to realize a controllable sequential compression response, convenient to realize multi-level protection.
(3) The layered gradient composite exhibited different response modes under different loading rates. Subjected to a low or medium loading rate (less than 10 m/s), the composite underwent an obvious layered response and negative Poisson’s effect, while it exhibited progressive collapse mode from the loading end subjected to a high loading rate (greater than 10 m/s).
(4) The composite with positive foam concrete density gradient (408:575:848) produced higher initial peak stress compared with that with negative gradient (848:575:408) by 21%, implying that the former was inferior to the latter in the aspect of initial transferred load to the protected structure.
(5) The effective Poisson’s ratio of the proposed composite first increased and then decreased, and finally remained stable with increasing compression stroke. Specifically, the effective Poisson’s ratio of the first layer in the composite with positive gradient (408:575:848) increased to −2 then converged to −0.6 under 2 m/s and 10 m/s crushing, and ranged from −0.4 to −0.1 under 50 m/s and 100 m/s crushing, respectively. The effective Poisson’s ratio of the middle and bottom layers increased to −2 initially and converged to range of −0.4 to −0.1, regardless of the crushing speed.

## 6. Further Research

The following aspects may be considered for future study:

(a) The theoretical model for auxetic honeycombs without and with filler may be established, to provide a quick preliminary prediction.
(b) Other configurations of hollow structures with auxeticity under compression may be investigated, especially with foam concrete filler.
(c) The theoretical model incorporating the blast load, the graded protective layer, and the protected structural member such as a beam or slab, may be established.

## Figures and Tables

**Table 1:** Material parameters of aluminum in the numerical model.

Material	Density/(kg/m^3^)	*E*/GPa	Poisson’s Ratio	Tangent Modulus/MPa
Aluminum	2700	68	0.36	31.25

**Table 2:** Material parameters of foam concrete in the numerical model.

Material	Density/(kg/m^3^)	*E*/MPa	Poisson’s Ratio	TSC/MPa	DAMP
Foam concrete-1	408	25	0.01	0.02	0.1
Foam concrete-2	575	52	0.01	0.1	0.1
Foam concrete-3	848	80	0.01	0.22	0.1

**Table 3:** The parameters of different specimens in the numerical simulation.

Specimen Index	Specimen Type	Cell-Wall Thickness Gradient	Average Cell-Wall Thickness/mm	Average Density/(kg/m^3^)
AH-0	Homogeneous	0.25:0.25:0.25	0.25	580
PGAH-1	Positive gradient	0:20:0:25:0.30	0.25	580
PGAH-2	Positive gradient	0:15:0:25:0.35	0.25	580
PGAH-3	Positive gradient	0:10:0:25:0.40	0.25	580
NGAH-1	Negative gradient	0.30:0.25:0.20	0.25	580
NGAH-2	Negative gradient	0.35:0.25:0.15	0.25	580
NGAH-3	Negative gradient	0.40:0.25:0.10	0.25	580

**Table 4:** The energy absorption of the foam concrete-filled honeycomb (*t* = 0.2:0.2:0.2 and *ρ* = 408:575:848, 1 m/s) in each compression stage.

Compression Stage	Nominal Strain	Displacement/mm	Energy/J	Proportion of Energy/%	Plateau Stress/MPa
Stage 1	0–0.216	0–29.16	243.41	16.51	1.16
Stage 2	0.216–0.45	29.16–60.74	520.94	35.33	1.42
Stage 3	0.45–0.662	60.74–89.37	710.04	48.16	2.58
Total	0–0.662	0–89.37	1474.39	100	-

**Table 5:** The energy absorption of the foam concrete filler at different compression stages (*t* = 0.2:0.2:0.2 and *ρ* = 408:575:848, 1 m/s).

Layer	Nominal Strain	Stage 1 Energy/J	Stage 1 Proportion/%	Stage 2 Energy/J	Stage 2 Proportion/%	Stage 3 Energy/J	Stage 3 Proportion/%
Top layer	0–0.216	187.72	94.62	36.18	8.51	23.44	3.75
Middle layer	0.216–0.45	9.32	4.70	377.41	88.72	58.16	9.31
Bottom layer	0.45–0.662	1.35	0.68	11.78	2.77	542.99	86.94
Total	0–0.662	198.39	100	425.37	100	624.59	100

## Data Availability

The data presented in this study are available on request from the corresponding author.
